# Complications of cochlear implantation: a decade’s experience

**DOI:** 10.1007/s00405-024-08855-y

**Published:** 2024-08-10

**Authors:** Badr Eldin Mostafa, Lobna El Fiky

**Affiliations:** Department of Otorhinolaryngology Head and Neck surgery, Faculty of Medicine, Ain-Sham,s University, 75 El Nozha Street 11351, Heliopolis-Cairo, Egypt

## Abstract

**Purpose:**

Surgery for cochlear implantation is becoming increasingly common. It is a precise surgery and carries with it a number of very specific complications. Although uncommon, they can profoundly affect the recipients’ quality of life. We report on our patients’ population and compare with different series.

**Methods:**

This is a retrospective analysis of patients who underwent cochlear implant surgery in our center or who were referred for management of complications between November 2012 and November 2022.

**Results:**

There were 2126 patients aged 9 months-68 years [mean 3.2 yrs] with 147 adults > 18 yrs. The male: female ratio was nearly 1. We are reporting on late complications excluding device failures. There were 186 complications [8.7%], 124 minor complications [66% of the complications, 5.8% of the total population; and 62 major complications [ 33% of the complications and 2.9% of the total]. The commonest minor complication was surgical site infection [16%] and the commonest major complication was flap necrosis and device extrusion [26%].

**Conclusion:**

Complications in our series were comparable to other series from different locations. But there seems to be a difference in the frequency of each complication depending on many factors which must be addressed. Standardization of reporting should be also more homogenized.

## Introduction

Cochlear implantation is becoming one of the routine practices in many otological centers with the number of implanted patients reaching more than 1 million worldwide [1]. Fortunately, the rate of complications seems to be quite small and improved significantly from the early series. [NO_PRINTED_FORM]with standardization of surgical techniques and the use of smaller and more biocompatible implants [2–4]. The global complication rate, varies around 12.5% [3.7–18.8%] with over two thirds being minor ones [Table 1]. These events seem related to various factors including the workload of the center, the proportion of adults and children, the number of surgeons and their experience and other undefined factors leading to differences in the rates and variety of complications. [5–11]

**Table 1:** Complication rates among various groups

Author	Year	Country	Number	Total %	Minor %	Major %	Commonest Minor	Commonest Major
(Binnetoglu et al., 2020)	2020	Turkey	2597	3.7	3	0.7	Vertigo	Extrusion
(Loundon et al., 2010)	2010	France	434	9.9	4.4	5.5	Vertigo SSI	Infection
(Castro et al., 2022)	2022	Brasil	432	15.5	12	5.4	Vertigo Headache SSI	Meningitis VII palsy
(Dagkiran et al., 2020)	2020	Turkey	1452	10.1	5.44	4.75	Hematoma Vertigo CTS AOM SSI	Flap Cholesteatoma Meningitis
(Rodolpho et al., 2010)	2010	Brasil	250	13.2	8	5.2	Vertigo SSI Tinnitus	Hematoma Infection
(Farinetti et al., 2014)	2014	France	403	19.9	14.9	5	Vertigo SSI CTS	Flap Meningitis Cholesteatoma
(Garrada et al., 2021)	2021	KSA	148	18.9	11.5	7.4	OME VII palsy	Flap necrosis Mastoiditis
(Sefein, 2018)	2018	Egypt	112	18.7	8.3	10.71	CSF leak	ASOM VII palsy
(Ahmed et al., 2021)	2021	Pakistan	251	6.4	4.8	1.6	AOM Pain Vertigo	Infection SSI
(AlKhtoum et al., 2022)	2022	Jordan	840	3.9			SSI Seroma	Flap necrosis
(Shahabaddin et al., 2024)	2024	Kurdistan	160	10	7.5	2.5	SSI VII palsy Nausea Headache	Extrusion Seroma
(Elfeky et al., 2021)	2021	Egypt	130	36	22	14	CTS SSI Hematoma Vertigo	VII CSF CSOM
(Mathews et al., 2023)	2023	India	1250	9	6	3		
(Ikeya et al., 2013)	2013	Japan	366		7.4	8.7		Flap infection
(Hansen et al., 2010)	2010	Denmark	367	29.1	27.3	1.8	Vertigo SSI CTS	SSI Hematoma
(Bhatia et al., 2004)	2004	UK	300	18.3	16	2.3	SSI Swelling Hematoma	Flap necrosis Chole CSOM
(Chiesa Estomba et al., 2017)	2017	Spain {adults}			17.5	24.6	Vertigo	Device failure
(Deep et al., 2021)	2021	US	136	14	11.2	2.9	Swelling OME ASOM	Wound breakdown Mastoiditis
(Halawani et al., 2019)	2019	KSA	1027	10.2	9.5	0.7	Swelling Seroma SSI Vertigo	Flap necrosis
(Brito et al., 2012)	2012	Brazil	550	16.7	7.8	8.9	VII palsy	Flap necrosis
(Rodolpho et al., 2010)	2010	Brazil	250	13.2	8	5.2	Vertigo SSI Tinnitus	Hematoma Infection
(Diab & Yusifov, 2018)	2018	Russia	967	4.2	2.6	1.6	Hematoma Vertigo AOM	Exposure CSOM
(Orlando & Cruz, 2024)	2024	Brazil	193		28	9.8	AOM SSI VII paresis Vertigo	SSI Hematoma

The aim of this study is to review ten years data of a tertiary referral cochlear implant center [ primary and referred cases] and compare our data with other studies from different geographical locations.

## Patients and methods

This is a retrospective analysis of patients who underwent cochlear implant surgery in our center or who were referred for management of complications between November 2012 and November 2022. There were 2126 patients aged 9 months-68 years [mean 3.2 yrs.] with 147 adults > 18 yrs. The ratio male: female was nearly 1. We are reporting on late complications excluding device failures. There were 186 complications [8.7%], 124 minor complications [66% of the complications, 5.8% of the total population; and 62 major complications [ 33% of the complications and 2.9% of the total] [8, 12]. One hundred and twenty patients were from our own patients and the remainder were referrals from other centers. [Table 2]. There were 850 MEDEL, 620 Cochlear, 524 AB and 132 Oticon implants. All cases were operated by a standard mastoidectomy/posterior tympanotomy technique. Ninety five patients had a postaurticular incision and 25 an extended endaural incision. Twenty-two patients had minimal hair clipping and the remainder a formal shave. Ethical committee approval was not required due to the retrospective nature of the study and anonymity of data.

**Table 2:** Complications in our series

	*N*	%	% of Total
**Minor**	**124**		**5.8**
Surgical site infection	30	24	16.1
Seroma	28	22.5	15.5
Chorda tympani syndrome	22	17.7	11.8
ASOM/OME	21	16.9	11.2
Temporary VII palsy	8	6.4	4.3
Vertigo	7	5.6	3.7
CSF leak	4	3.2	2.1
Facial twitches	2	1.6	1
**Major**	**62**		**2.9**
Flap infection and necrosis	49	79	26.3
Cholesteatoma	7	11.2	3.7
Tympanic membrane perforation	4	6.45	2.1
Meningitis	2	3.22	1
Permanent Facial palsy	2	3.22	1

**Table 3:** Comparison between our average complications rate and the series’ averages

	Minimum	Maximum	Range	Mean	Median	SD	Our series
Total	2	36	34	13.36	13	8.24	**8.7**
Minor	2.6	28	25.4	11.05	8.15	7.05	**5.8**
Major	0.7	24.6	23.9	6.01	5.1	5.5	**2.9**

### Minor complications

- Wound infection [SSI] [30] :
  - Superficial skin infection with dehiscence not involving the deeper tissues or exposing the device. Usually treated by local dressings and oral antibiotic. All cases healed uneventfully within 10 days.
- Hematoma/Seroma: [28]
  - Delayed 1 month/5 years. May be attributed to minor trauma or (?) allergic reaction or subclinical infection. Most cases were treated conservatively [ Compression; Antibiotics/Steroids/Antihistamines; Aspiration under aseptic conditions and compression].
  - Seven cases progressed to frank infection, flap necrosis and device loss.
- Chorda tympani dysfunction: [22]
  - All cases did not have surgically reported injury. Adults and older children were specifically asked 3 months after surgery for taste disturbances. In 5 patients the symptom was troublesome and affected their QOL.
- ASOM/OME: [21]
  - Medically treated uneventfully.
- Temporary facial palsy [8].
  - Treated by steroids and recovering within a month.
- Transient vertigo [7].
  - Mostly of short duration especially in adults and in bilateral cases. It was persistent in only two adults > 1 year. Children were tested by the Bruininks-Oseretsky Test of Motor Proficiency (BOT2) 24–48 h after surgery. Adults with persistent vertigo were subjected to vHIT, rotatory test and VNG to rule out other possible causes. [13–15]Management is by vestibular suppressants for the first couple of days. In persistent cases vestibular rehabilitation was successful in one case and in the other a single IT gentamycin injection led to complete cessation of the vertigo without affecting the implant performance.
- Perilymph / CSF leak [4].
  - In patients with inner ear malformations [common cavity 1; IP I ,1 ;IP II,2 ]. Three were managed conservatively by compression, acetazolamide and/or mannitol. One underwent lumbar drainage once.
- Facial twitches [2].
  - One patient had a transient period of facial twitching 2 months after surgery which stopped spontaneously. Another had progressive twitches with the use of the device. CT scan revealed rarefaction at the level of the middle turn and possible contact between the lateral wall electrode and the facial nerve. The device was replaced by an Advanced bionics MS electrode and the problem was resolved.

### Major complications

- Flap necrosis [49].
  - This was the most problematic complication. The time between implantation and flap problems ranged between 1 week and 5 years. The progression from redness, crust formation, skin loss, infection and exposure of the device varied widely between patients. In some it was a rapidly progressive inexorable process [16]. In others it was an indolent event with remissions with treatment and exacerbations. The vast majority were children < 3 yrs [32 cases], 5 adults and 12 > 8 years ].
  - In 22 cases the mean time between surgery and flap problems was 9 months, in the remaining cases it was more than 1 year. In 43 cases, the device was exposed and the wound infected at presentation. Local dressings and antibiotics were instituted to control infection and the device was explanted and the wound debrided and closed by local rotation flaps. In 6 cases local dressings and antibiotics were used and after subsidence of infection local rotation flaps were used to cover the device and salvage it. This was only successful in 2 cases and in the remainder the device had to be explanted. Of the 47 explanted cases, 35 were immediately implanted on the opposite side, 7 were implanted on the opposite side later and 5 cases reimplanted on the same side after 6–9 months.
- Cholesteatoma [7].
  - One case had a simple perforation during the first surgery and was primarily grafted. He developed a cholesteatoma 3 years after surgery. The other cases were referred from other centers with persistent discharge and in one case extrusion of the electrode through the external canal. All cases underwent explantation and subtotal petrosectomy. In 5 cases the electrode was cut short at the level of the round window and kept as a stent.
- Tympanic membrane perforation [4].
  - Two patients had an attack of acute suppurative otitis media which was treated medically and resolved but left a residual perforation. One patient did not report any history of infection and the third patient had a traumatic perforation after ear wash. All underwent myringoplasty [2 temporalis fascia and two tragal cartilage].
- Permanent Facial paralysis [2].
  - Both were referred cases and reported immediate postoperative facial paralysis. One had a cut at the level of the tympanic portion. The ear was explored and the nerve grafted by a greater auricular graft. The other had an exposure at the lower end of the posterior tympanotomy and pressure by the electrode. The nerve was grossly intact so it was decompressed and the electrode displaced away from the nerve and fascia was interposed.
- Meningitis [2].
  - One case had an attack of fever and mild disturbance of consciousness after an episode of acute otitis media 2 months after surgery. She was hospitalized and recovered under antibiotic treatment. The second case was an IP I case who underwent subtotal petrosectomy and obliteration during the primary surgery. He developed two attacks of meningitis despite this. Review of the radiology revealed that the other side was also abnormal and was suspected to be the source of infection. Subtotal petrosectomy and obliteration were performed, and the patient did not have any further attacks.

## Discussion

Cochlear implantation is becoming a common surgical procedure with increasing indications involving patients at the extremes of age, patients with complex syndromes and inner ear malformations [1, 17–20]. Fortunately, the rate of complications is quite low when we exclude device failures which are not directly related to the procedure as such [9, 21, 22]. Depending on the perspective, complications can be classified as early or late, minor, or major [9, 13, 21]. The overall rate and proportion of minor vs. major complications seems to be universal across different series. However, there are some differences between the type and frequency of complications. This seems related to various factors including the workload of the center, the proportion of adults and children and other undefined factors leading to differences in the rates and variety of complications [20, 23–32]. 

In our series, the most frequent late complication was flap infection and necrosis with device loss [2.3% of the total and nearly 48% of the major complications]. We reviewed all these cases to try to elucidate any possible risk factors. There were no differences between a minimal post auricular and extended endaural incisions, no difference between deep and shallow beds, no difference between hair shaving and minimal hair clipping. There was a significantly higher proportion in children < 3 years compared to older children or adults. In two adults, the device was not in a subperiosteal pocket but rather intramuscular. In three patients, one child and two adults, uncontrolled diabetes was the possible comorbid factor. There were no significant differences between different brands. [7, 22, 32–36]

In contrast to some studies, the incidence of vestibular problems was much lower in our series (3.7%). This may be due to the higher proportions of pediatric patients who usually compensate quite rapidly and don’t suffer from vertigo or instability, and may not report [8, 9, 13, 14, 21, 23, 26, 31, 32, 37].

Chorda tympani affection varies widely between studies and seems to be underreported especially in pediatric series. In our series the incidence seems to be high as we questioned patients directly rather than self-reporting. [5, 9, 29, 38, 39]

The commonest major complications in most series seems to be flap infection, device extrusion and facial paralysis [5, 11, 26, 30, 32, 37, 40, 41], much less common are cholesteatoma, CSF leaks, meningitis, mastoiditis and facial palsy [21, 29, 31, 42, 43]. The commonest minor complication seems to be vertigo followed by acute otitis media/otitis media with effusion, temporary CSF leak and chorda tympani dysfunction [11, 26, 29, 31, 32, 37, 44]. Few authors report on pain or headache, nausea and vomiting, tinnitus while others do not. This may lead to a spurious increase in their rate of complication [31, 33, 36, 40].

Although classification of complications has been more or less standardized, reporting is still not consistent and what a group considers a major complication, another considers it minor depending on the management policy or the stringency of interpretation. [8, 21, 45, 46]

The differences between different centers around the world seem insignificant, this is an indicator that cochlear implant surgery has become well standardized. Table 3 Surgeon related complications are extremely uncommon and mostly minor and/or temporary [31, 38, 40]. An important factor seems to be the close supervision and strict rules implemented by different implant manufacturers in establishing different programs [5, 6, 21, 23, 44]. Infections, seroma and hematoma are the commonest late complications. A number of measures were suggested to minimize infections including vaccinations, intra and postoperative antibiotics, early management of acute otitis media and/or otitis media with effusion [24, 25, 28, 40, 47]. Improvement in complications rates needs more comprehensive studies and address the commonest ones especially flap problems, infections and device extrusion. Improvement in device designs, magnet pressure on the soft tissues and possibly the shell material of the devices [1]. Long term follow-up is also mandatory as some complications may be delayed for years [7, 21, 32, 41, 44, 48, 49].
